# Can Risk Stratification Based on Ultrasound Elastography of Background Liver Assist CEUS LI-RADS in the Diagnosis of HCC?

**DOI:** 10.3389/fonc.2021.662680

**Published:** 2021-04-30

**Authors:** Jiawu Li, Wenwu Ling, Shuang Chen, Lulu Yang, Lin Ma, Qiang Lu, Yan Luo

**Affiliations:** Department of Ultrasound, West China Hospital, Sichuan University, Chengdu, China

**Keywords:** contrast-enhanced ultrasound, liver imaging reporting and data system, liver, hepatocellular carcinoma, ultrasound elastography

## Abstract

**Objective:**

To explore whether risk stratification based on ultrasound elastography of liver background assists contrast-enhanced ultrasound liver imaging reporting and data system (CEUS LI-RADS) in diagnosing HCC.

**Materials and Methods:**

In total, 304 patients with focal liver lesions (FLLs) confirmed by pathology underwent CEUS and ultrasound elastography were included in this retrospective study. Patients with chronic hepatitis B (CHB, n=193) and non-CHB (n=111) were stratified by four liver stiffness measurement (LSM) thresholds. A LI-RADS category was assigned to FLLs using CEUS LI-RADS v2017. The diagnostic performance was assessed with the AUC, sensitivity, specificity, PPV, and NPV.

**Results:**

The mean background liver stiffness of HCC patients with CHB, HCC patients without CHB and non-HCC patients without CHB were 9.72 kPa, 8.23 kPa and 4.97 kPa, respectively. The AUC, sensitivity, specificity and PPV of CEUS LI-RADS for HCC in CHB patients with LSM ≥ 5.8 kPa, ≥ 6.8 kPa, ≥ 9.1 kPa, and ≥ 10.3 kPa were high, with corresponding values of 0.745 to 0.880, 94.2% to 95.3%, 81.3% to 85.7%, and 98.1% to 98.8%, respectively. Higher AUC and specificity for HCC was observed in non-CHB patients with LSM ≥ 9.1 kPa and ≥ 10.3 kPa compared to non-CHB patients with LSM ≥ 5.8 kPa and ≥ 6.8 kPa, with corresponding values of0.964/1.000 vs 0.590/0.580, and 100%/100% vs 60%/70%, respectively.

**Conclusion:**

CEUS LI-RADS has a good diagnostic performance in CHB patients regardless of the background liver stiffness. Furthermore, CEUS LI-RADS can be applied for non-CHB patients with a LSM ≥ 9.1 kPa.

## Introduction

Hepatocellular carcinoma (HCC) is the fifth most common malignant tumor in the world and the third leading cause of cancer death (1, 2). The main causes of liver cancer include liver cirrhosis, chronic viral hepatitis, nonalcoholic fatty hepatitis, chronic alcoholism and autoimmune hepatitis (3, 4). The treatment and prognosis of early liver cancer completely differ from that of advanced liver cancer, and the median survival time of advanced liver cancer patients is often less than one year (5). Therefore, the early diagnosis and treatment of liver cancer are very important for patient prognosis.

Imaging surveillance for high-risk populations with HCC is recommended by many major liver disease societies worldwide (6–8). Although the importance of noninvasive imaging in diagnosing HCC has been emphasized, there are no unified, widely accepted diagnostic algorithms or standards for this disease. The contrast-enhanced ultrasound liver imaging reporting and data system (CEUS LI-RADS) was initially developed by the American College of Radiology (ACR) in 2016 to solve this problem. LI-RADS was mainly developed for populations with high-risk factors for HCC, and several studies have reported that this system has good diagnostic accuracy (9–15). Furthermore, the latest guidelines and recommendations for contrast-enhanced ultrasound of the liver published by the World Federation for Ultrasound in Medicine and Biology (WFUMB) indicate that CEUS LI-RADS can be utilized to establish a diagnosis of malignancy (CEUS LR-M) or, specifically, HCC (CEUS LR-5), in high-risk patients (16). However, the identification of high-risk factors for HCC may be challenging in clinical practice (17). In most cases, radiologists can determine whether patients have high-risk factors for HCC through laboratory examination. However, identifying high-risk patients only by conventional imaging can sometimes be difficult for radiologists because radiologists do not always receive complete clinical information about patients to determine whether they have risk factors for HCC, especially first-time outpatients and patients in developing countries. As ultrasound is a first-line screening and diagnostic method for liver cancer, the following question arises: is there any way to help ultrasound physicians determine whether a patient is at risk for HCC?

Ultrasound elastography is a novel ultrasound diagnostic technique that has been recommended by several guidelines for use in clinical practice (18–20), and this modality is a very useful tool for the noninvasive detection of the degree of liver fibrosis (21–23). However, no studies have reported whether liver stiffness can be used to stratify patients or how liver stiffness can be used to stratify patients with or without risk factors for HCC; the role of liver stiffness in complementing the CEUS LI-RADS is also worthy to be explored. Therefore, this study will explore the feasibility of using liver stiffness measured by ultrasound elastography to stratify patients with unknown risk factors for HCC and evaluate the diagnostic performance of the CEUS LI-RADS v2017 guidelines (24) for HCC in different groups.

## Materials and Methods

### Patient Selection

**Figure 1:**
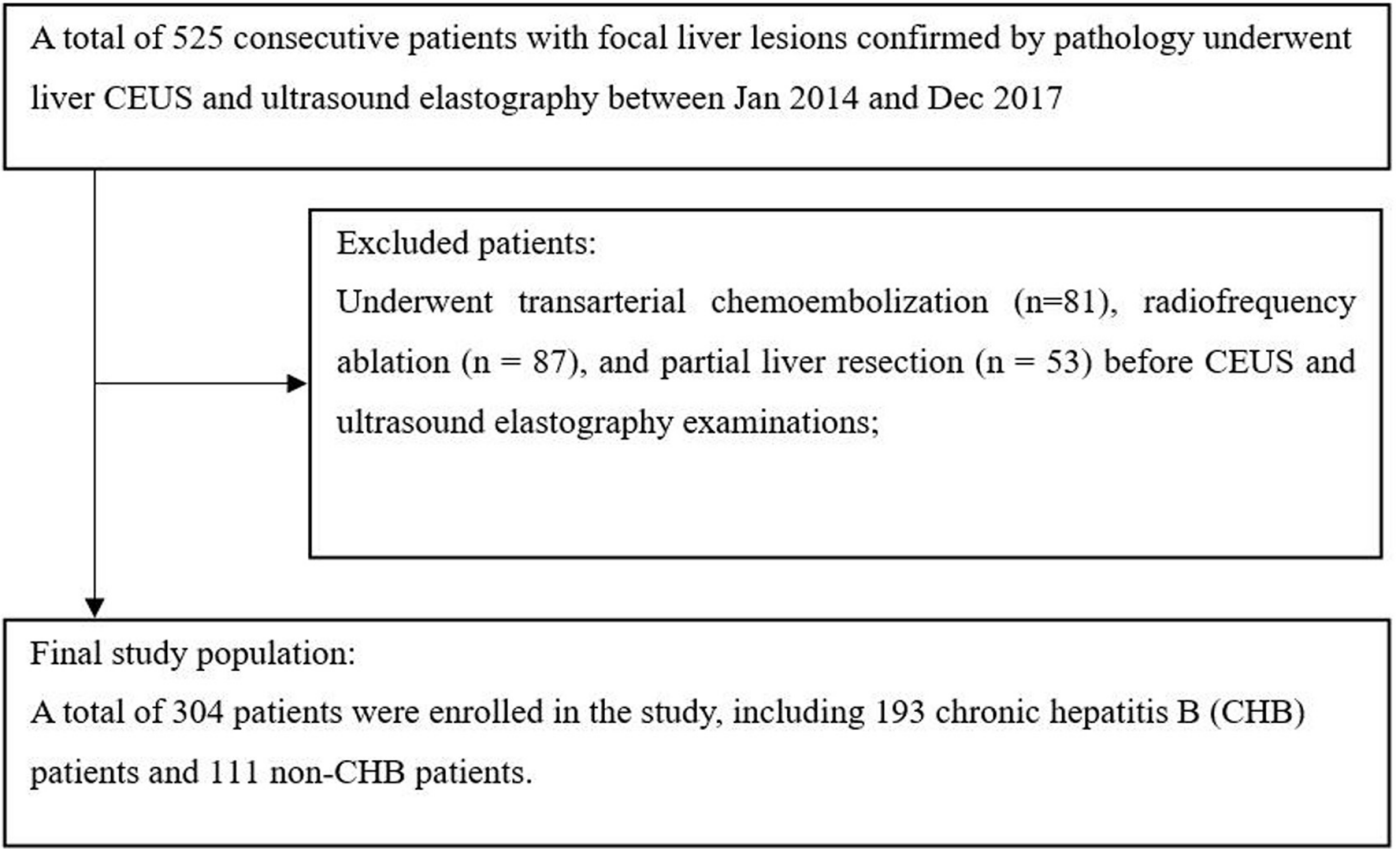
The flow chart for inclusion of patients in the study.

This study was approved by the ethics committee of West China Hospital in Sichuan University, and the requirement for written informed consent from all subjects was waived. Between January 2014 and December 2017, patients who underwent liver CEUS and ultrasound elastography were consecutively included in this retrospective study. The inclusion criteria were as follows: (1) both CEUS and ultrasound elastography examinations were performed; and (2) the type of focal liver lesions (FLL) was confirmed by pathology. Patients were excluded if they had hepatic lesions treated with clinical intervention (such as radiofrequency ablation, hepatic arterial chemoembolization, or partial liver resection) before CEUS and ultrasound elastography. A total of 525 patients with FLLs confirmed by pathology underwent both liver CEUS and ultrasound elastography. Two hundred twenty-one patients with focal liver lesions who underwent clinical intervention before CEUS and ElastPQ were excluded. **Figure 1** shows the patient inclusion flow chart for the study.

### Liver Stiffness Measurements (LSM)

Ultrasound elastography was performed with an iU22 ultrasound system (Royal Philips, the Netherlands) equipped with a C5-1 (1–5 MHz) transducer (mechanical index 1.1), which is a shear wave-based technology to assess the stiffness properties of the liver. The examinations were conducted by an ultrasound physician (J.L.) with more than 3 years of experience in ultrasound elastography examinations. All ultrasound elastography examinations were performed on the same machine and half an hour after CEUS examinations. All patients fasted before ultrasound examinations. The liver stiffness measurement (LSM, expressed in kilopascals, kPa) was performed 5 times in a vessel-free area of the background liver in the right lobe. Finally, the mean value of 5 measurements within an individual was used in the statistical analysis.

### CEUS Examinations

On the same day of the ultrasound elastography examinations, CEUS examinations were performed by two ultrasound physicians (Y.L. and Q.L., each with more than 10 years of experience in diagnosing liver diseases) using the same ultrasound system and transducer as those used to measure liver stiffness, with a real-time, low-mechanical index (MI: 0.05-0.08), reverse pulse imaging technique. In total, 2.4 mL of the SonoVue ultrasound contrast agent (Bracco, Milan, Italy) was injected into the medial cubital vein and immediately flushed with 5 mL 0.9% sodium chloride solution. A timer was immediately started after the contrast agent was injected, and a dynamic digital video of the target lesion and surrounding liver parenchyma within the first minute was stored continuously for analysis. After 60 seconds, intermittent scanning was performed to prevent the microbubbles from being destroyed too quickly; scanning continued until the microbubbles were completely cleared, and typical contrast-enhanced images of the lesions were stored for each scan.

### Imaging Analysis

Two readers independently categorized the patients according to the ACR CEUS LI-RADS v2017 guidelines (24). The two readers (S.C. and W.L.) have 7 and 8 years of experience with CEUS. The dynamic digital video within the first minute, typical contrast-enhanced images at rest in the portal phase and late phase, conventional ultrasound images and the diameter of the target lesion were provided to both readers. Both readers were blinded to the clinical information, ultrasound elastography results and final diagnostic reports of each patient. In cases with a discrepancy, an experienced ultrasound physician who has 15 years of experience in CEUS decided the final category. For patients with more than one hepatic lesion, the most visible and clear lesion was chosen for analysis. Treated lesions were not included in the study.

### Risk Stratification of Non-CHB Patients by Liver Stiffness Measured With Ultrasound Elastography

Ultrasound elastography can be used to effectively differentiate the degree of liver fibrosis. In our clinical practice (22), using liver stiffness (F1: 5.8 kPa, F2: 6.8 kPa, F3: 9.1 kPa, and F4: 10.3 kPa) measured by ultrasound to diagnosis liver fibrosis has a good diagnostic performance (areas under the curve (AUCs) from 0.87 to 0.97). In the study, CHB patients and non-CHB patients were stratified into four subgroups by the cutoff value of each fibrosis stage.

### Tissue Sampling

Tissue specimens of the lesion were obtained by biopsy or surgical resection. Biopsy was performed by using an 18-gauge core needle with ultrasound-guided puncture. The liver background of the biopsied or surgical specimen was staged by using Scheuer fibrosis staging.

### Statistical Analysis

MedCalc (10.4.7.0) and SAS 9.4 statistical software were used to perform data analysis. Differences were considered significant at *P*<0.05. The Mann-Whitney unpaired test was used to evaluate the differences in tumor size, background liver stiffness, and age distribution between CHB patients and non-CHB patients. Categorical variables were compared with the χ^2^ test. The sensitivity, specificity, positive predictive value (PPV) and negative predictive value (NPV) of the CEUS LI-RADS for HCC were calculated in each group using LI-RADS category 5 as diagnostic criteria for positive HCC. The diagnostic accuracy of LI-RADS was estimated by calculating the area under the receiver operating characteristic (ROC) curve (AUC).

## Results

### Patient and Tumor Characteristics

A total of 304 patients (238 men, 66 women) were enrolled in the study, including 193 chronic hepatitis B (CHB) patients and 111 non-CHB patients. The baseline characteristics of the patients are shown in **Table 1**. Surgical resection was performed for 292 FLLs, and ultrasound-guided biopsy was performed for the other 12 FLLs. The mean age ± standard deviation was 50.6 ± 11.6 years for the CHB group and 53.5 ± 13.5 years for the non-CHB group. The mean tumor size (SD, range) in the CHB group was 5.0 (3.4, 1.1∼17) cm, which was significantly smaller than that of the non-CHB group (5.6 (3.4, 1.5∼17) cm) (*P* = 0.013). The mean background liver stiffness of the CHB group (9.6 ± 3.2 kPa) was significantly greater than that of the non-CHB group (6.3 ± 2.7 kPa) (*P* = 0.000). In addition, in the group of non-CHB patients, there were 32 cases of chronic hepatitis C, six cases of autoimmune hepatitis, two cases of alcohol-related liver disease, five cases of schistosomiasis infection, seven cases of nonalcoholic steatohepatitis (NASH), and 59 cases of unknown risk factors. None of the non-CHB patients were diagnosed with cirrhosis before surgery.

**Table 1:** Baseline characteristics of study patients.

Variables	CHB patients (n=193)	Non-CHB patients (n=111)	*P* value
**Age (mean±SD,year)**	50.6 ± 11.6	53.5 ± 13.5	0.015
**Male**	162 (83.9%)	76 (68.5%)	0.003
**Female**	31 (16.1%)	35 (31.5%)	
**Tumor size (mean±SD, cm)**	5.0 ± 3.4	5.6 ± 3.4	0.013
**Background liver stiffness (mean±SD, kPa)**	9.6 ± 3.2	6.3±2.7	0.000
**Child-Pugh stage**			0.198
A	184	109	
B	9	2	
**Pathology confirmed lesions & background liver**			
**HCC**	173	41	0.000
Fatty	1 (0.6%)	4 (9.7%)	
Fibrotic	60(34.7%)	29 (70.7%)	
Cirrhotic	112 (64.7%)	8 (19.5%)	
Normal	0	0	
**Non-HCC malignancies**	16	41	0.000
Fatty	3 (18.7%)	4 (9.8%)	
Fibrotic	11 (68.8%)	5 (12.2%)	
Cirrhotic	2 (12.5%)	0	
Normal	0	32 (78%)	
**Benign lesion**	4	29	0.012
Fatty	1 (25%)	3 (10.3%)	
Fibrotic	0	1 (3.4%)	
Cirrhotic	2 (50%)	0	
Normal	1 (25%)	25 (86.2%)	

SD, standard deviations; cm, centimeter; CHB, chronic hepatitis B; kPa, kilopascal; HCC, hepatocellular carcinoma. Unless otherwise stated, data are numbers of patients, with percentage in parentheses.

In the group of CHB patients, the final diagnoses for all FLLs were as follows: 173 (89.1%, 173/193) were diagnosed with HCC, 16 (8.3%, 16/193) were diagnosed with non-HCC malignancies (nine with intrahepatic cholangiocarcinoma, four with liver metastasis, two with combined hepatocellular-cholangiocarcinoma, and one with sarcomatoid hepatocellular carcinoma), and 4 (2.6%, 4/193) were diagnosed with benign lesions (two with hyperplastic nodules and two with hepatic inflammatory lesions). A total of 64.7% of CHB patients with HCC had cirrhosis, and 34.7% had different degrees of liver fibrosis. In the group of non-CHB patients, 41 (36.9%, 41/111) were diagnosed with HCC, 41 (36.9%, 41/111) were diagnosed with non-HCC malignancies (27 with intrahepatic cholangiocarcinoma, 12 with liver metastasis, one with sarcomatoid hepatocellular carcinoma, and one with primary liver neuroendocrine carcinoma), and 29 (26.1%, 29/111) were diagnosed with benign lesions (16 with focal nodular hyperplasia (FNH), four with hepatic abscess, three with hepatic adenoma, three with hepatic angiomyolipoma, and three with hepatic paragonimiasis). The majority of HCC patients in the non-CHB group had different degrees of liver fibrosis (70.7%, 29/41), while 19.5% (8/41) had cirrhosis. Most patients with non-HCC malignancies (78%, 32/41) and benign lesions (86.2%, 25/29) in the non-CHB group had a normal liver background, which was significantly different from that in the CHB group (**Table 1**).

### Distribution of Background Liver Stiffness in Different Patients

**Figure 2:**
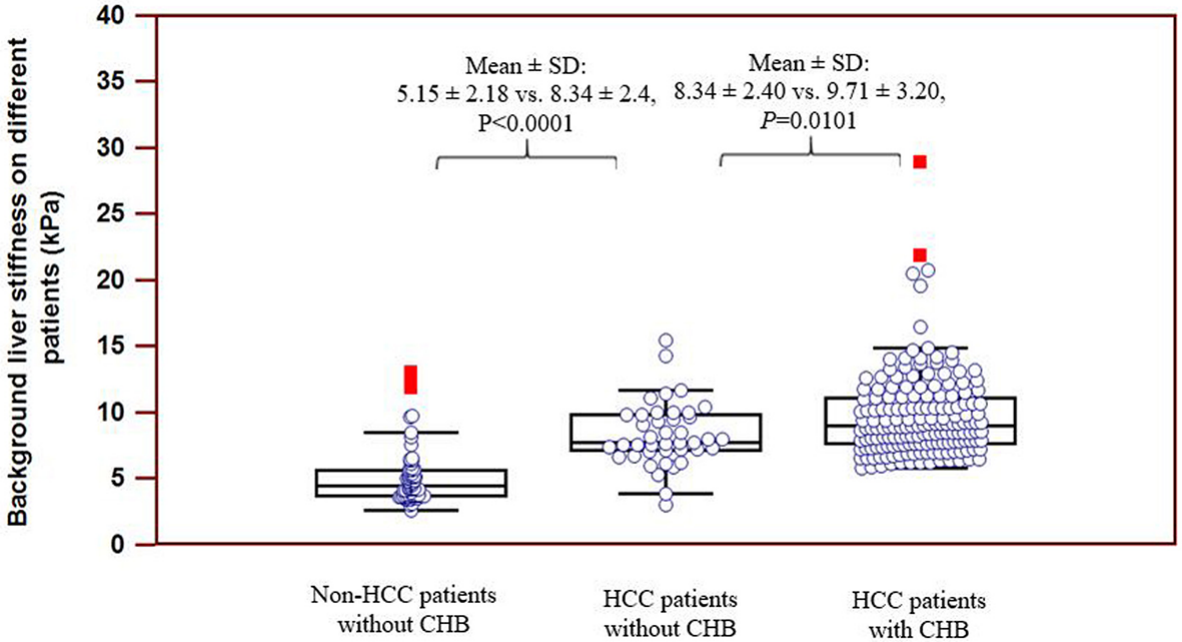
Distribution of background liver stiffness on different patients. Significantly higher stiffness value was found on HCC patients without CHB as compared to non-HCC patients without CHB (*P* < 0.0001). Significantly higher stiffness value was found on HCC patients with CHB as compared to HCC patients without CHB (*P* = 0.0101).

In the group of CHB patients, 60.1% (116/193) of the patients had cirrhosis, and 36.8% (71/193) had different degrees of liver fibrosis, while in the group of non-CHB patients, only 7.2% (8/111) had cirrhosis, and 31.5% (35/111) had different degrees of liver fibrosis. The distributions of background liver stiffness in non-HCC patients without chronic hepatitis B (CHB) and HCC patients with CHB and non-CHB are presented in **Figure 2**. The mean (SD) background liver stiffness for HCC patients with CHB was 9.72 (3.19) kPa. The mean (SD) background liver stiffness for HCC and non-HCC patients without CHB was 8.23 (2.44) kPa and 4.97 (2.05) kPa, respectively. A significantly higher liver stiffness value was found in HCC patients without CHB than in non-HCC patients without CHB (*P* < 0.0001). A significantly higher liver stiffness value was found in HCC patients with CHB than in HCC patients without CHB (*P* = 0.0101).

### CEUS LI-RADS Categories in the Group of CHB Patients and Non-CHB Patients

The LI-RADS categories of the lesions in the group of CHB patients and non-CHB patients are summarized in **Table 2**. In the group of CHB patients, none of the LI-RADS category 3 lesions, 6 (100%) category 4 lesions, 163 (97.6%) of 167 category 5 lesions, and 4 (21.1%) of 19 category M lesions were HCCs. Three (1.8%) of 167 category 5 lesions and 13 (68.4%) of 19 category M lesions were non-HCC malignancies. In the non-CHB group, no patient was diagnosed with cirrhosis before surgery, so CEUS LI-RADS was not directly applicable to patients in the group.

**Table 2:** Distribution of CEUS LI-RADS in the group of CHB and non-CHB patients (without using liver stiffness).

LI-RADS category	CHB patients (n=193)			Non-CHB patients (n=111)		
	HCC	Non-HCC malignancies	Benign Lesions	HCC	Non-HCC malignancies	Benign Lesions
LR-3	0	0	1 (100%)	NA	NA	NA
LR-4	6 (100%)	0	0	NA	NA	NA
LR-5	163 (97.6%)	3 (1.8%)	1 (0.6%)	NA	NA	NA
LR-M	4 (21.1%)	13 (68.4%)	2 (10.5%)	NA	NA	NA

The table presented the distribution of CEUS LI-RADS in the group of CHB and non-CHB patients. In the non-CHB group, no patient was diagnosed with cirrhosis before surgery, so CEUS LI-RADS was not directly applicable to patients in the group. CEUS, contrast enhanced ultrasound; LI-RADS, Liver Imaging Reporting and Data System; HCC, hepatocellular carcinoma; CHB, chronic hepatitis B; NA, not applicable.

### In-Depth Analysis of CEUS LI-RADS Categories in the Group of CHB Patients and Non-CHB Patients With Different Liver Stiffness Thresholds

**Figure 3:**
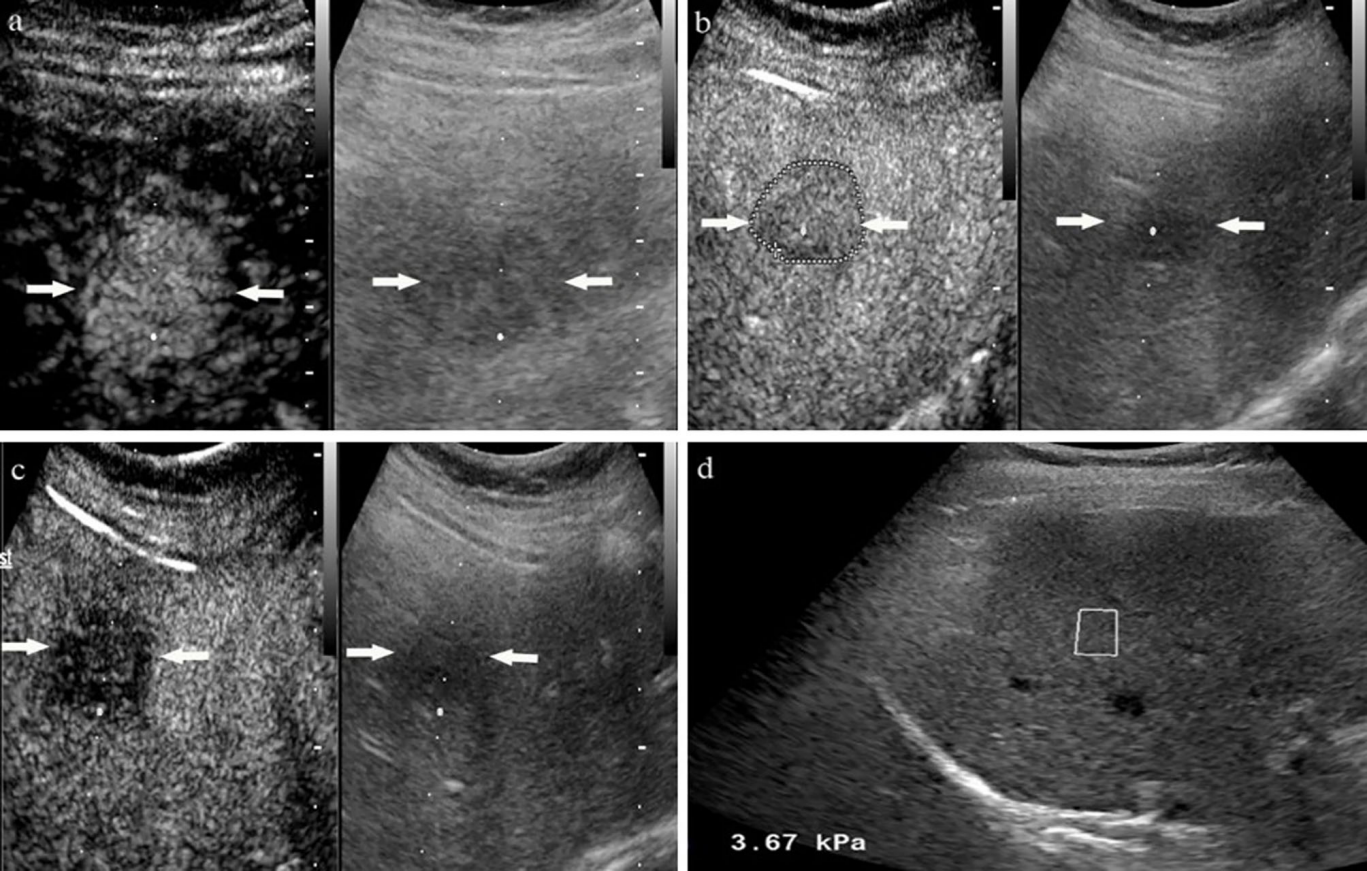
A 54-year-old woman with non-chronic hepatitis B virus complained of right epigastric discomfort. A hypoechoic tumor with largest diameter of 2.8 centimeters in anterior segment of the liver. At the arterial phase of contrast enhanced ultrasound, the mass showed rapidly whole hyperenhancement **(A)**, and began washout after 60 seconds **(B)**, finally with slightly washout at late phase **(C)**. Liver stiffness measurement of liver background by ultrasound elastography showed the liver stiffness was 3.67 kPa **(D)**. The lesion proved to be inflammatory pseudotumor by pathology.

**Figure 4:**
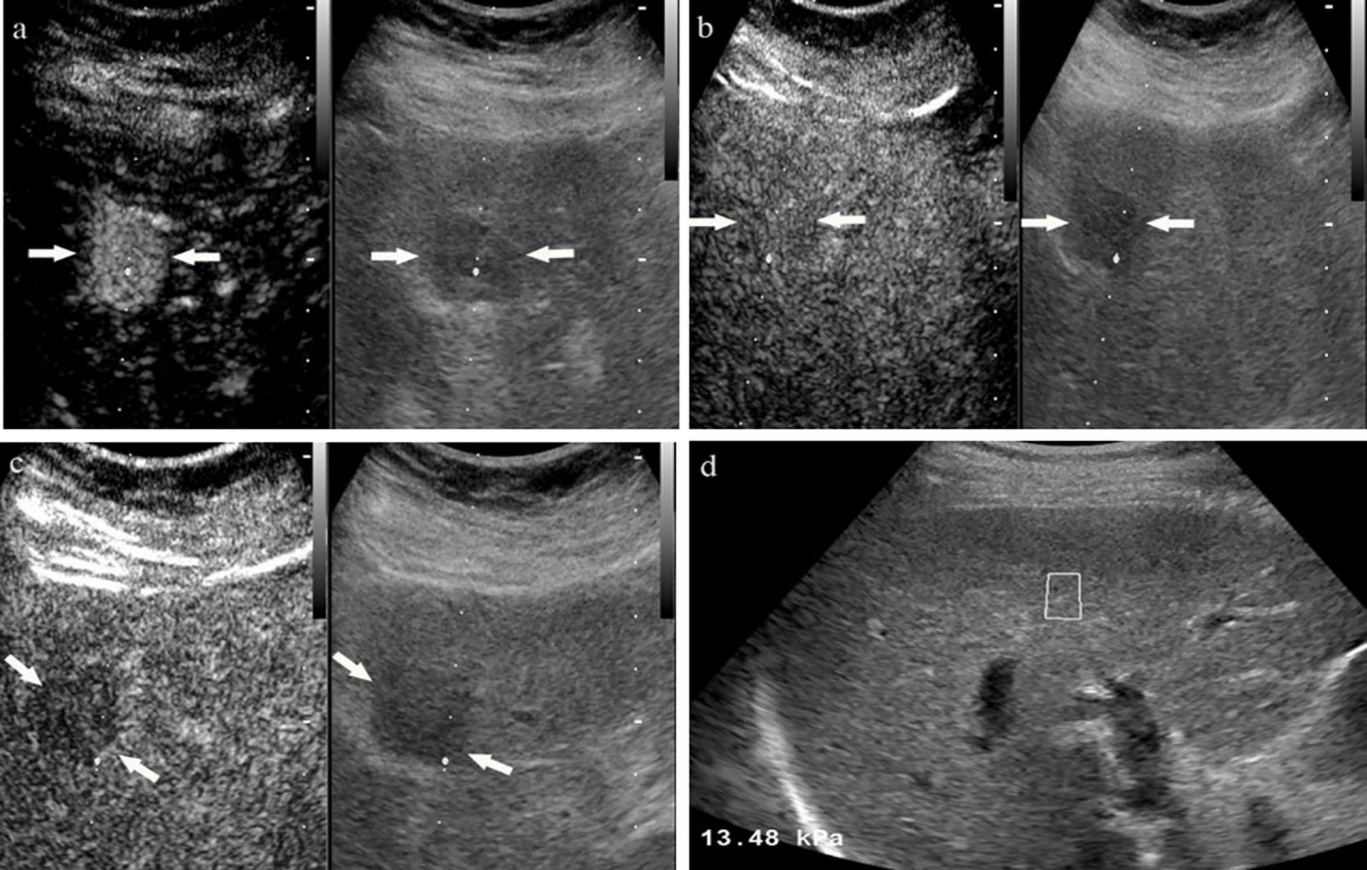
A 58-year-old male with unknown risk factors for HCC. The arrow highlights a 2.4 cm hypoechoic tumor in liver segment IV. At the arterial phase of contrast enhanced ultrasound, the mass showed rapidly whole hyper-enhancement **(A)**, and presented as iso-enhancement at portal phase **(B)**, finally with slightly washout at late phase **(C)**. Ultrasound elastopraphy showed the stiffness value of liver background was 13.48 kPa **(D)**. The lesion was proved to be moderately differentiated hepatocellular carcinoma by pathology.

The LI-RADS categories of the lesions in the subgroup of CHB patients and non-CHB patients with different liver stiffness thresholds are presented in **Table 3**. The percentages of category 5 HCC lesions in the CHB patients with LSM ≥ 5.8 kPa, LSM ≥ 6.8 kPa, LSM ≥ 9.1 kPa, and LSM ≥ 10.3 kPa were 98.2%, 98.6%, 98.8%, and 98.1%, respectively. In the non-CHB patients, the proportion of HCC in category 5 increased with the increase in liver stiffness threshold, from 82.9% to 100% (**Figures 3** and **4**).

**Table 3:** Distribution of CEUS LI-RADS in the group of CHB and non-CHB patients with different liver stiffness thresholds.

Group	Pathology	LR-3	LR-4	LR-5	LR-M
**CHB patients**					
LSM≥5.8 kPa	HCC	0	6 (100%)	164 (98.2%)	4 (25%)
(n=190)	Non-HCC malignancies	0	0	2 (1.2%)	11 (68.8%)
	Benign Lesions	1 (100%)	0	1 (0.6%)	1 (6.2%)
LSM≥6.8 kPa	HCC	0	6 (100%)	146 (98.6%)	3 (27.3%)
(n=166)	Non-HCC malignancies	0	0	1 (0.7%)	7 (63.6%)
	Benign Lesions	1 (100%)	0	1 (0.7%)	1 (9.1%)
LSM≥9.1 kPa	HCC	0	4 (100%)	81 (98.8%)	0
(n=92)	Non-HCC malignancies	0	0	1 (1.2%)	6 (100%)
	Benign Lesions	0	0	0	0
LSM≥10.3 kPa	HCC	0	3 (100%)	53 (98.1%)	0
(n=62)	Non-HCC malignancies	0	0	1 (1.9%)	5 (100%)
	Benign Lesions	0	0	0	0
**Non-CHB patients**					
LSM≥5.8 kPa	HCC	0	2 (66.7%)	29 (82.9%)	6 (46.2%)
(n=52)	Non-HCC malignancies	0	0	1 (2.8%)	7 (53.8%)
	Benign Lesions	1 (100%)	1 (33.3%)	5 (14.3%)	0
LSM≥6.8 kPa	HCC	0	2 (66.7%)	25 (89.3%)	4 (44.4%)
(n=41)	Non-HCC malignancies	0	0	1 (3.6%)	5 (55.6%)
	Benign Lesions	1 (100%)	1 (33.3%)	2 (7.1%)	0
LSM≥9.1 kPa	HCC	0	2 (66.7%)	13 (100%)	1 (33.3%)
(n=20)	Non-HCC malignancies	0	0	0	2 (66.7%)
	Benign Lesions	1 (100%)	1 (33.3%)	0	0
LSM≥10.3 kPa	HCC	0	0	7 (100%)	0
(n=9)	Non-HCC malignancies	0	0	0	1 (100%)
	Benign Lesions	1 (100%)	0	0	0

This table showed the distribution of CEUS LI-RADS categories in CHB patients and non-CHB with different liver stiffness thresholds. CEUS, contrast enhanced ultrasound; LI-RADS, Liver Imaging Reporting and Data System; HCC, hepatocellular carcinoma; LS, liver stiffness; kPa, kilopascal. CHB, chronic hepatitis B; Note. Data are numbers of patients, data in parentheses are percentages.

### Diagnostic Performance of CEUS LI-RADS for HCC in the Group of CHB Patients and Non-CHB Patients With Different Liver Stiffness Thresholds

As presented in **Table 4**, when considering LI-RADS category 5 as HCC, the area under the curve (AUC), sensitivity, specificity and PPV for HCC in CHB patients were 0.816, 94.3%, 84.2%, and 98.2%, respectively.

**Table 4:** Diagnostic performance of CEUS LI-RADS for HCC in the group of CHB and non-CHB patients with different liver stiffness thresholds.

Group	LR-5 as diagnostic criteria for HCC				Diagnostic Performance				
	TP	FP	FN	TN	Sensitivity	Specificity	PPV	NPV	AUC
**CHB patients**									
Overall	164	3	10	16	94.3%	84.2%	98.2%	61.5%	0.816
LSM≥5.8 kPa	164	3	10	13	94.3%	81.3%	98.2%	56.5%	0.786
LSM≥6.8 kPa	146	2	9	9	94.2%	81.8%	98.6%	50.0%	0.745
LSM≥9.1 kPa	81	1	4	6	95.3%	85.7%	98.8%	60.0%	0.880
LSM≥10.3 kPa	53	1	3	5	94.6%	83.3%	98.1%	62.5%	0.865
**Non-CHB patients**									
Overall	32	19	8	52	80.0%	73.2%	62.7%	86.7%	0.611
LSM≥5.8 kPa	29	6	8	9	78.4%	60.0%	82.9%	52.9%	0.590
LSM≥6.8 kPa	25	3	6	7	80.6%	70.0%	89.3%	53.8%	0.580
LSM≥9.1 kPa	13	0	3	4	81.3%	100%	100%	57.1%	0.964
LSM≥10.3 kPa	7	0	0	2	100%	100%	100%	100%	1.000

CEUS, contrast enhanced ultrasound. LI-RADS, Liver Imaging Reporting and Data System; HCC, hepatocellular carcinoma; LS, liver stiffness; kPa, kilopascal. CHB, chronic hepatitis B; TP, true-positive; FP, false-positive; FN, false-negative; TN, true-negative; PPV, positive predictive value; NPV, negative predictive value; AUC, area under the curve.

The AUC, sensitivity, specificity and PPV for HCC in CHB patients with LSM ≥ 5.8 kPa, LSM ≥ 6.8 kPa, LSM ≥ 9.1 kPa, and LSM ≥ 10.3 kPa were high, with corresponding values of 0.786, 94.3%, 81.3% and 98.2% vs 0.745, 94.2%, 81.8% and 98.6% vs 0.880, 95.3%, 85.7% and 98.8% vs 0.865, 94.6%, 83.3% and 98.1%, respectively.

CEUS LI-RADS showed poor diagnostic performance in non-CHB patients, with an AUC, specificity and PPV of 0.611, 73.2% and 62.7%, respectively. However, CEUS LI-RADS showed good diagnostic performance in non-CHB patients with LSM ≥ 9.1 kPa and ≥ 10.3 kPa, and the AUC, sensitivity, specificity and PPV for HCC were 0.964/1.000, 81.3%/100%, 100%/100%, and 100%/100%, respectively. However, the AUC, sensitivity, and specificity of CEUS LI-RADS for HCC were moderate in non-CHB patients with LSM ≥ 5.8 kPa and 6.8 kPa, with corresponding values of 0.590/0.580, 78.4%/80.6%, and 60%/70%, respectively. Higher diagnostic performance of CEUS LI-RADS for HCC was observed in non-CHB patients with LSM ≥ 9.1 kPa and LSM ≥ 10.3 kPa compared to non-CHB patients with LSM ≥ 5.8 kPa and LSM ≥ 6.8 kPa.

## Discussion

Identifying high-risk factors for hepatocellular carcinoma (HCC) can be challenging in clinical practice. This study explored whether risk stratification based on ultrasound elastography of the liver background of chronic hepatitis B patients (CHB) and non-CHB patients assists CEUS LI-RADS in diagnosing HCC. The results showed that the diagnostic performance of CEUS LI-RADS was good in CHB patients with different degrees of liver stiffness, which indicated that CHB patients, even without high liver stiffness values, are still at risk for HCC. In addition, for those non-CHB patients, CEUS LI-RADS had good diagnostic performance at LSM ≥ 9.1 kPa and 10.3 kPa and moderate at LS ≥ 5.8 kPa and LS ≥ 6.8 kPa.

In this study, we explored the relationship between background liver stiffness and focal liver lesion nature in different patients. The results showed that the liver stiffness of CHB patients with HCC was significantly higher than that of non-CHB patients with HCC, which may be related to the fact that hepatitis B is a major factor leading to liver cancer, and long-term inflammatory stimulation would lead to liver fibrosis and cirrhosis. However, most HCC patients have a liver background of fibrosis or cirrhosis, regardless of whether the patients have chronic hepatitis B or not. Furthermore, in non-CHB patients, the background liver stiffness of HCC patients was significantly higher than that of non-HCC patients, which suggests that non-CHB patients with higher liver stiffness may have a greater risk for HCC. Masuzaki et al. (25) reported that liver stiffness measurements can be a predictor of HCC in patients with hepatitis C virus infections. However, whether increased liver stiffness increases the risk of HCC in other populations warrants further exploration. Furthermore, approximately 20% of HCC cases have been known to develop in a noncirrhotic liver (26). Therefore, attempts can be made to stratify non-CHB and noncirrhotic patients to identify patients with underlying fibrosis or cirrhosis, which is conducive to further consideration of benign and malignant lesions in imaging diagnosis.

The majority of LR-4 and LR-5 lesions (100% and 97.6%, respectively) in CHB patients were HCC in the study, which is almost in line with the study of Zheng et al. (15). Meanwhile, for CHB patients, the proportion of HCC lesions in category 5 (from 98.1% to 98.8%) was high regardless of the liver stiffness threshold. In addition, the study showed that for non-CHB patients, the proportion of HCC in category 5 increased with the increase in liver stiffness threshold, from 82.9% to 100%. The results may indicate that with the increase in liver stiffness, the probability of HCC is greater, which needs a larger sample size for further study. It is worth mentioning that among the HCC patients without chronic hepatitis B, 70.7% (29/41) had liver fibrosis, while only 19.5% (8/41) had cirrhosis in the study. Therefore, this suggests that more attention may be paid to patients with liver fibrosis in nonhepatitis B patients.

We separately analyzed the diagnostic performance of CEUS LI-RADS for HCC in different liver stiffness thresholds of CHB patients and non-CHB patients. For CHB patients, the diagnostic accuracy, sensitivity, specificity and PPV of CEUS LI-RADS for HCC were high among each subgroup of different liver stiffness. The results confirmed that CEUS LI-RADS is suitable for CHB patients regardless of their background liver stiffness. The results showed that the diagnostic performance of CEUS LI-RADS for HCC in the group of non-CHB patients was poor, while the diagnostic performance was good in non-CHB patients with LSM ≥ 9.1 kPa and ≥ 10.3 kPa. In addition, the diagnostic performance of CEUS LI-RADS for HCC was poor in non-CHB patients with LSM ≥ 5.8 kPa and ≥ 6.8 kPa. The results showed that using liver stiffness measured by ultrasound elastography to stratify non-CHB patients and further evaluated by CEUS LI-RADS can be helpful. However, validations on larger cohorts should be further performed due to the limitation on the number of study subjects included in the study.

There were some limitations in our study. First, the number of patients in each group was relatively small, and validations on large cohorts should be performed. Second, only one kind of ultrasound elastography was used in this study, and the generalizability of these results to other kinds of ultrasound elastography needs to be further validated. Third, the stiffness properties of FLLs were not measured in the study, so whether the elasticity of FLLs combined with CEUS LI-RADS contributes to the diagnosis of HCC was not further evaluated. Finally, to strengthen the reference standard, only patients with pathological confirmation were included, which also led to patient selection bias in the study.

In conclusion, this study illustrated that using liver stiffness measured by ultrasound elastography to stratify non-CHB patients was feasible. CEUS LI-RADS can be directly applied to CHB patients regardless of their liver stiffness and non-CHB patients with LSM ≥ 9.1 kPa.

## Data Availability Statement

The raw data supporting the conclusions of this article will be made available by the authors, without undue reservation.

## Ethics Statement

The studies involving human participants were reviewed and approved by Ethics committee of West China Hospital in Sichuan University. Written informed consent for participation was not required for this study in accordance with the national legislation and the institutional requirements.

## Author Contributions

Study design: All authors. Data Collection: JL. Data analysis and interpretation: JL, WL, SC, LM, LY, QL, and YL. Manuscript writing: JL. All authors contributed to the article and approved the submitted version.

## Funding

This research was supported by National Natural Science Foundation of China (No. 81701702 and 82001832).

## Conflict of Interest

The authors declare that the research was conducted in the absence of any commercial or financial relationships that could be construed as a potential conflict of interest.
